# Poisson noisy image restoration via overlapping group sparse and nonconvex second-order total variation priors

**DOI:** 10.1371/journal.pone.0250260

**Published:** 2021-04-20

**Authors:** Kyongson Jon, Jun Liu, Xiaoguang Lv, Wensheng Zhu

**Affiliations:** 1 Key Laboratory for Applied Statistics of MOE, School of Mathematics and Statistics, Northeast Normal University, Changchun, P.R. China; 2 Faculty of Mathematics, Kim Il Sung University, Pyongyang, D.P.R. of Korea; 3 School of Science, Jiangsu Ocean University, Lianyungang, Jiangsu, P.R. China; Chongqing University, CHINA

## Abstract

The restoration of the Poisson noisy images is an essential task in many imaging applications due to the uncertainty of the number of discrete particles incident on the image sensor. In this paper, we consider utilizing a hybrid regularizer for Poisson noisy image restoration. The proposed regularizer, which combines the overlapping group sparse (OGS) total variation with the high-order nonconvex total variation, can alleviate the staircase artifacts while preserving the original sharp edges. We use the framework of the alternating direction method of multipliers to design an efficient minimization algorithm for the proposed model. Since the objective function is the sum of the non-quadratic log-likelihood and nonconvex nondifferentiable regularizer, we propose to solve the intractable subproblems by the majorization-minimization (MM) method and the iteratively reweighted least squares (IRLS) algorithm, respectively. Numerical experiments show the efficiency of the proposed method for Poissonian image restoration including denoising and deblurring.

## 1 Introduction

In many real applications, during the image recording, the measurement of light inevitably leads to the uncertainty of striking particles on the image sensor. In other words, the finite number of electrons or photons carrying energy in an image sensor may cause statistical fluctuations, which are usually modeled as the Poisson distribution [1–3]. As another degradation factor, image formation may involve undesirable blurrings such as motion or out-of-focus. By rewriting the concerned images into column-major vectorized form, we can regard the observed image $g\in R^{n\times n}$ as a realization of Poisson random vector with expected value *Hf* + *b*, where *H* is a *n*^2^ × *n*^2^ convolution matrix corresponding to the point spreading function (PSF) which models blur effects, $f\in R^{n\times n}$ is the original image, and $b\in R^{n\times n}$ is a nonnegative constant background [4–6].

Poissonian image restoration calls for applying an inverse procedure to approximate *f* from an observation *g* degraded by blur and Poisson noise. A general option to tackle this problem is to use the maximum a posteriori (MAP) estimation from Bayesian perspective. Taking the Poisson statistics of noise into account, we can write the conditional distribution of the observed data *g* as

$$\begin{array}{c} p\left(g|f\right)=\prod_{i,j=1}^{n}\frac{e^{-{\left(Hf+b\right)}_{i,j}}{\left(Hf+b\right)}_{i,j}^{g_{i,j}}}{g_{i,j}!}, \end{array}$$ (1)

where (⋅)_*i*,*j*_ indicates a vector element corresponding to position (*i*, *j*) hereafter. On the premise that we adopt a Gibbs prior [7–9] of the form

$$\begin{array}{c} p\left(f\right)\propto e^{-\phi (f)/\lambda } \end{array}$$ (2)

for *f*, the use of Bayes’ rule and Stirling’s approximation leads to the following minimization problem [10, 11]:

$$\begin{array}{c} \underset{f}{\text{min}}\{\lambda D_{\text{KL}}\left(Hf||g\right)+\phi \left(f\right)\}, \end{array}$$ (3)

where λ > 0 is a regularization parameter, *ϕ*(*f*) is a nonnegative given function (often called the regularizer), and *D*_KL_(*Hf*||*g*) denotes the generalized Kullback-Leibler (KL) divergence of *Hf* from *g* as shown below:

$$\begin{array}{c} D_{\text{KL}}\left(Hf||g\right)≔\sum_{i,j=1}^{n}[{\left(Hf+b\right)}_{i,j}-g_{i,j}+g_{i,j}\text{log}\frac{g_{i,j}}{{\left(Hf+b\right)}_{i,j}}]. \end{array}$$ (4)

Since the efficiency of the restoration model hinges on the choice of image regularizer, numerous authors have proposed different regularization methods. Tikhonov regularization of form $\phi \left(f\right)=\|\Gamma f{\|}_{2}^{2}$ (usually, *Γ* is an identity matrix or difference matrix) is probably one of the most classical methods. In the literatures, several methods have been proposed to efficiently solve Eq (3) with the Tikhonov regularizer, including the scaled gradient projection method [12], the split-gradient method [13], the projected Newton-conjugate gradient (PNCG) method [2], the quasi-Newton projection method [14], the hybrid gradient projection-reduced Newton method [15, 16] and the scaled gradient projection-type method [17]. Although the KL divergence and Tikhonov regularizer are well-known to be convex and the unique solution to Eq (3) is guaranteed with inexpensive computational cost, it tends to over-smooth the important details in restored images.

Another classical method, the total variation (TV) regularizer *ϕ*(*f*) = ‖*f*‖_TV_ (see Section 2), has been widely accepted since the noisy signals have larger TV than the original signal [18]. Bardsley et al. [19] proved that minimizing the Poisson likelihood function in conjunction with TV regularization is well-posed, and they also verified its convergence by a nonnegatively constrained and projected quasi-Newton minimization algorithm. Landi et al. [20] also adopted the TV regularizer for denoising the medical images collected by the photon-counting devices and extended the PNCG method introduced in [2]. Tao et al. [21] appended a non-negativity constraint and used the half-quadratic splitting method to solve the related minimization problem. In addition to the methods mentioned above, other authors employed the effective optimization techniques for solving the TV regularized Poisson deblurring model, including the alternating extra-gradient method [22], the primal-dual method [23, 24] and the alternating direction method of multipliers (ADMM) [25, 26]. It is well-known that the TV regularization methods could preserve fine details such as sharp edges, but they often exhibit false jump discontinuities causing spurious staircase artifacts in smooth regions of the restored images. This may be due to the fact that the TV regularization tends to transform the smooth regions of the solution into piecewise constant ones while solving the minimization problem.

One possible remedy for this oversharpening behavior is to introduce high-order TV, which could penalize jumps more. Zhou et al. [27] selected the second-order TV *ϕ*(*f*) = ‖*f*‖_HTV_ (see Section 2) as a regularizer, and solved by using the alternating minimization scheme. Their numerical experiments indicated the advantage of the second-order TV in avoiding the staircase artifacts, compared with the classical TV regularization [28]. But the high-order TV usually transforms the smooth signal into over-smoothing [29], so the hybrid regularizations combining the high-order TV with other regularizers were also considered. Among the hybrid models, Zhang et al. [30] obtained better results than the model with a strongly convex term ${\left\|f\right\|}_{2}^{2}$ proposed in [31], by replacing the first-order TV with a second-order TV. Besides, Jiang et al. [11] combined the first-order and second-order TV priors to restore images contaminated by Poisson noise and solved the minimization model by the ADMM. To further improve the restoration result, Liu et al. [5] studied a spatially adapted regularization parameter updating scheme. As an adaptive balancing scheme between the first and second derivatives, Wang et al. [32] established the Poisson noise removal framework of iterative reweighted total generalized variation (TGV) model based on the EM algorithm for the denoising case. Zhang et al. [33] combined the fractional-order TV with non-local TV to alleviate the staircase artifacts for the cartoon component as well as to preserve the details for the texture component. Ma et al. [34] proposed a hybrid regularizer containing a patch-based sparsity promoting prior over a learned dictionary and a pixel-based total variation prior, but it requires additional strategies to reduce the high computational cost.

Considering that the tight framelet framework can facilitate the sparse representation of images [35], Shi et al. [36] combined the framelet regularization with non-local TV and non-negativity constraint in the non-blind stage of blind Poisson deblurring. Zhang et al. [37] proposed the nonconvex and noncontinuous model with *ℓ*_0_ norm of the tight wavelet representation as a regularizer. Fang and Yan [38] combined the *ℓ*_1_ norm of framelet coefficients with TV for Poissonian image deconvolution to reduce artifacts yielded by only using TV regularization. In addition to working with transform-domain sparsity mentioned above, the overlapping group sparsity, which describes the natural tendency of large values to arise near other large values rather than in isolation, has been widely concerned in the field of image restoration, due to its remarkable ability to exploit the structural information of the natural image gradient [4, 39–41]. Especially, for the Poisson noisy restoration, Lv et al. [4] focused on the regularization by the total variation with overlapping group sparsity (TVOGS) and they showed that the solution obtained by the ADMM framework is superior to the first-order TV regularized methods [25, 42] and the high-order regularized methods [27].

To sum up, the high-order TV or the overlapping group sparse prior can be the most feasible option to alleviate the staircase artifacts. Nonetheless, the high-order TV, due to the over-penalizing tendency of the *ℓ*_1_ norm [43], often causes over-smoothing at the edges while the overlapping group sparsity tends to smoothen out blockiness in the restored images more globally. Adam and Paramesran, motivated by the work of Chen et al. [44], proposed a hybrid regularization model that combined the overlapping group sparse total variation with the high-order nonconvex TV to denoise images corrupted by Gaussian noise [40]. Recently, they extended it to non-blind deblurring under Gaussian noise [45]. In this paper, we present an extension of Adam’s regularizer to the problem of restoring the Poisson noisy images. This regularizer takes the advantages of both the high-order nonconvex regularizer and overlapping group sparsity regularizer, i.e., it can simultaneously facilitate the pixel-level and structured sparsities of the natural image in the gradient domain. Therefore, we expect that it can not only effectively reduce staircase artifacts but also preserve well sharp edges in the restored image. However, its optimization is more challenging than Gaussian deblurring due to the ill-conditioned non-quadratic data fidelity term. We employ the alternating direction method of multipliers to solve the derived minimization problem. In particular, during the ADMM iterations, we solve two intractable subproblems: one is from overlapping group sparse prior and solved by majorization-minimization (MM) method with well-known quadratic majorizer; another is from the nonconvex *ℓ*_*p*_(0 < *p* < 1) quasi norm, which is solved by the iteratively reweighted least squares (IRLS) algorithm with the motivation that the IRLS is guaranteed to converge to its local minima provided better theoretical and practical abilities than the iteratively reweighted *ℓ*_1_ (IRL1) algorithm [52–55].

The rest of this paper is organized as follows. In Section 2, we introduce some notations that will be used to formulate our proposed method. We also briefly review the essential concepts and tools including the overlapping group sparsity prior, the ADMM algorithm, the MM method, and the IRLS algorithm. Section 3 establishes a novel Poissonian image deblurring model which comprises the generalized Kullback Leibler (KL) divergence as data fidelity term, and a combined first-order and second-order total variation priors with overlapping group sparsity as a regularization term. Sequentially, we derive an effective algorithm for minimizing the non-convex and non-smooth objective function under the ADMM optimization framework. Section 4 demonstrates the superiority of our method via numerical experiments, followed by analyzing the parameter setting and convergence behavior. We finally conclude this paper in Section 5.

## 2 Preliminaries

### 2.1 Notations

It is necessary to introduce some notations throughout this paper. Assuming that all images under consideration are gray-scale images of size *n* × *n*, we lexicographically stack the columns of an image matrix into a vector form. For example, the (*i*, *j*)th pixel of image *f* becomes the ((*j* − 1)*n* + *i*)th entry of the vector, just written as *f*_*i*,*j*_. Under the periodic boundary conditions for image *f*, we introduce the discrete forward difference operator $D^{+}:R^{n\times n}\to (R^{n\times n},R^{n\times n})$ defined by

$$\begin{array}{c} (D^{+}f{)}_{i,j}≔((D_{1}^{+}f{)}_{i,j},(D_{2}^{+}f{)}_{i,j})\in R^{2}, \end{array}$$ (5)

and similarly for the backward difference operator $D^{-}:R^{n\times n}\to \left(R^{n\times n},R^{n\times n}\right)$,

$$\begin{array}{c} (D^{-}f{)}_{i,j}≔((D_{1}^{-}f{)}_{i,j},(D_{2}^{-}f{)}_{i,j})\in R^{2}, \end{array}$$ (6)

where the definitions of each forward and backward sub-operators are separately:

$$\begin{array}{c} (D_{1}^{+}f{)}_{i,j}≔\{\begin{array}{cc} f_{i,j+1}-f_{i,j}, & \text{if}\;j<n\\ f_{i,1}-f_{i,n}, & \text{if}\;j=n \end{array}\;,\;(D_{2}^{+}f{)}_{i,j}≔\{\begin{array}{cc} f_{i+1,j}-f_{i,j}, & \text{if}\;i<n\\ f_{1,j}-f_{n,j}, & \text{if}\;i=n \end{array}, \end{array}$$

$$\begin{array}{c} (D_{1}^{-}f{)}_{i,j}≔\{\begin{array}{cc} f_{i,j}-f_{i,j-1}, & \text{if}\;j>1\\ f_{i,1}-f_{i,n}, & \text{if}\;j=1 \end{array}\;,\text{and}\;(D_{2}^{-}f{)}_{i,j}≔\{\begin{array}{cc} f_{i,j}-f_{i-1,j}, & \text{if}\;i>1\\ f_{1,j}-f_{n,j}, & \text{if}\;i=1 \end{array}. \end{array}$$ (7)

Second-order difference operators can be recursively defined by using the first-order difference operators such as

$$\begin{array}{c} (D_{11}^{-+}f{)}_{i,j}≔(D_{1}^{-}(D_{1}^{+}f){)}_{i,j}, \end{array}$$ (8)

and other second-order difference operators can be similarly defined. Based on the above definitions, we denote the first and second-order TV of *f* as

$$\begin{array}{c} \|f{\|}_{\text{TV}}≔\sum_{i,j=1}^{n}\|{\left(\nabla f\right)}_{i,j}{\|}_{2}\;and\;\|f{\|}_{\text{HTV}}≔\sum_{i,j=1}^{n}\|{\left(\nabla^{2}f\right)}_{i,j}{\|}_{2}, \end{array}$$ (9)

where ‖⋅‖_2_ means the Euclidean norm, ${\left(\nabla f\right)}_{i,j}=((D_{1}^{+}f{)}_{i,j},(D_{2}^{+}f{)}_{i,j}),\text{and}\;{\left(\nabla^{2}f\right)}_{i,j}=((D_{11}^{-+}f{)}_{i,j},(D_{12}^{++}f{)}_{i,j},(D_{21}^{++}f{)}_{i,j},(D_{22}^{-+}f{)}_{i,j})$.

### 2.2 TVOGS and MM algorithm

To describe the structural sparsity of image gradient, we define a pixel-group ${\tilde{\text{v}}}_{(i,j),K}$ of size *K* × *K* (*K* is called group size) centered at every position (*i*, *j*) of a two-dimensional array **v** = (*v*_*i*,*j*_)_*n* × *n*_ as

$$\begin{array}{c} {\tilde{\text{v}}}_{(i,j),K}=[\begin{array}{cccc} v_{i-m_{1},j-m_{1}} & v_{i-m_{1},j-m_{1}+1} & \cdots & v_{i-m_{1},j+m_{2}}\\ v_{i-m_{1}+1,j-m_{1}} & v_{i-m_{1}+1,j-m_{1}+1} & \cdots & v_{i-m_{1}+1,j+m_{2}}\\ \vdots & \vdots & \ddots & \vdots \\ v_{i+m_{2},j-m_{1}} & v_{i+m_{2},j-m_{1}+1} & \cdots & v_{i+m_{2},j+m_{2}} \end{array}]\in R^{K\times K}, \end{array}$$ (10)

where $m_{1}=\left\lfloor \frac{K-1}{2}\right\rfloor ,m_{2}=\left\lfloor \frac{K}{2}\right\rfloor$, and ⌊⋅⌋ denotes the floor function, i.e., it converts any real number into a nearest integer less than or equal to it. Let *v*_(*i*,*j*),*K*_ be the column-major vectorized form of ${\tilde{\text{v}}}_{(i,j),K}$, i.e., $v_{(i,j),K}={\tilde{\text{v}}}_{(i,j),K}\left(:\right)$. Then, as in Liu et al.’s work [46], we can denote the TVOGS regularizer *ϕ*_TO_(*f*) as

$$\begin{array}{c} \phi_{\text{TO}}\left(f\right)≔\phi_{O}\left(\nabla_{1}f\right)+\phi_{O}\left(\nabla_{2}f\right), \end{array}$$ (11)

where $\nabla_{1}f≔D_{1}^{+}f$ and $\nabla_{2}f≔D_{2}^{+}f$ denote the horizontal and vertical gradient of *f*, respectively, and $\phi_{O}\left(v\right)=\sum_{i,j=1}^{n}\|v_{(i,j),K}{\|}_{2}$ is the *K*-group OGS function of $v\in R^{n^{2}}$. For the notational simplicity, we denote *ϕ*_O_(∇*f*) = *ϕ*_O_(∇_1_ *f*) + *ϕ*_O_(∇_2_ *f*).

Next, we review a minimization problem of the form

$$\begin{array}{c} \underset{v}{\text{min}}\{\frac{1}{2}\|v-v_{0}{\|}_{2}^{2}+\lambda \phi_{O}\left(v\right)\}, \end{array}$$ (12)

and we denote the objective function of Eq (12) as *R*(*v*).

To solve the above sophisticated OGS model, the majorization-minimization approach is used with $\psi (v|u)=\frac{1}{2}\sum_{i,j=1}^{n}[\frac{1}{\|u_{(i,j),K}{\|}_{2}}\|v_{(i,j),K}{\|}_{2}^{2}+\|u_{(i,j),K}{\|}_{2}]$ as a quadratic majorizer of *ϕ*_O_(*v*), namely, *ψ*(*v*|*u*) ≥ *ϕ*_O_(*v*) for all *v* and *u* ≠ **0** and with equality if *v* = *u*. Therefore, instead of the intractable direct optimization of *R*(*v*), we can approximately solve it through iterative minimizing a sequence of surrogate convex problems:

$$\begin{array}{c} \begin{array}{cc} v^{\left(k+1\right)} & =\text{arg}\;\underset{v}{\text{min}}Q(v|v^{\left(k\right)}),k=0,1,\cdots ,\\ Q & (v|v^{\left(k\right)})=\frac{1}{2}\|v-v_{0}{\|}_{2}^{2}+\lambda \psi (v|v^{\left(k\right)})=\|v-v_{0}{\|}_{2}^{2}+\lambda \|\Lambda (v^{\left(k\right)})v{\|}_{2}^{2}, \end{array} \end{array}$$ (13)

where Λ(*u*) is a diagonal matrix containing the following entries along its diagonal

$$\begin{array}{c} {}[\Lambda (u){]}_{l,l}=\sqrt{\sum_{i,j=-m_{1}}^{m_{2}}[\sum_{k_{1},k_{2}=-m_{1}}^{m_{2}}|u_{r-i+k_{1},t-j+k_{2}}{|}^{2}{]}^{-\frac{1}{2}}} \end{array}$$ (14)

with *l* = (*r* − 1)*n* + *t*, for *r*, *t* = 1, 2, ⋯, *n*. Eq (13) has the closed-form solution

$$v^{\left(k+1\right)}=(I_{n^{2}}+\lambda \Lambda {\left(v^{\left(k\right)}{)}^{T}\Lambda (v^{\left(k\right)})\right)}^{-1}v_{0},$$ (15)

which is the input for the next MM iteration. The inversion of the matrix $I_{n^{2}}+\lambda \Lambda {\left(v^{\left(k\right)}\right)}^{T}\Lambda (v^{\left(k\right)})$ can be efficiently done via simple component-wise calculations. In summary, we obtain the following algorithm for Eq (12).

**Algorithm 1** MM algorithm for minimizing Eq (12)

**input** λ > 0, *K*, *N*_in_(inner iterations)

**initialize** *v*^(0)^ = *v*_0_, *k* = 0.

**while** *k* < *N*_in_ **do**             ▹ MM inner loop

 1: $[\Lambda (v^{\left(k\right)}){]}_{l,l}=\sqrt{\sum_{i,j=-m_{1}}^{m_{2}}[\sum_{k_{1},k_{2}=-m_{1}}^{m_{2}}|v_{r-i+k_{1},t-j+k_{2}}^{\left(k\right)}{|}^{2}{]}^{-\frac{1}{2}}}$;

 2: $v^{\left(k+1\right)}={\left(I_{n^{2}}+\lambda \Lambda {\left(v^{\left(k\right)}\right)}^{T}\Lambda (v^{\left(k\right)})\right)}^{-1}$ *v*_0_;

 3: *k* = *k* + 1;

**end while**

### 2.3 Alternating direction method of multipliers

The standard form of the ADMM [47] is designed to tackle the distributed convex optimization problem with linear equality constraints, and many variants have been developed [48–50]. Recently, Wang et al. [51] extended it to the problems involving the nonconvex and nonsmooth muti-blocks objective, as follows:

$$\begin{array}{c} \begin{array}{cc} & \min_{x,y}\sum_{i=1}^{s}F_{i}\left(x_{i}\right)+G\left(y\right)\\ & \;\text{subject}\;\text{to}\;\sum_{i=1}^{s}A_{i}x_{i}+By=0, \end{array} \end{array}$$ (16)

where $x=\left[x_{1};\cdots ;x_{s}\right]\in R^{N}$ (here $x_{i}\in R^{n_{i}},N=\sum_{i=1}^{s}n_{i}$) and $y\in R^{L}$ are primal variables with the corresponding coefficient matrix $A_{i}\in R^{M\times n_{i}}$ and $B\in R^{M\times L}$ respectively, $F_{i}:R^{n_{i}}\to R$ is a continuous function, and $G:R^{L}\to R$ is a smooth function.

In general, $F_{i}$ can be nonsmooth and nonconvex, and G can be nonconvex. By introducing a Lagrangian multiplier $w\in R^{M}$ and a penalty parameter *δ* > 0 for the linear constraint $\sum_{i=1}^{s}A_{i}x_{i}+By=0$, we obtain the augmented Lagrangian in a scaled form,

$$\begin{array}{c} L_{\delta }\left(x,y,w\right)=\sum_{i=1}^{s}F_{i}\left(x_{i}\right)+G\left(y\right)+\frac{\delta }{2}\|\sum_{i=1}^{s}A_{i}x_{i}+By+\frac{w}{\delta }{\|}_{2}^{2}. \end{array}$$ (17)

The ADMM algorithm proceeds to alternatively update each variable (*x*^(*k*+1)^, *y*^(*k*+1)^) until it reaches a stationary point of Eq (17) or meets a stopping criterion. Then, we have the following iterative scheme:

$$\begin{array}{c} \begin{array}{cc} x_{1}^{\left(k+1\right)} & \in \text{arg}\;\min_{x_{1}}F_{1}\left(x_{1}\right)+\frac{\delta }{2}\|\;A_{1}x_{1}+A_{2}x_{2}^{\left(k\right)}+\cdots +A_{s}x_{s}^{\left(k\right)}+By^{\left(k\right)}+\frac{w^{\left(k\right)}}{\delta }{\|}_{2}^{2};\\ \;\;\;\vdots \; & \;\;\;\vdots \;\;\;\;\vdots \\ x_{s}^{\left(k+1\right)} & \in \text{arg}\;\min_{x_{s}}F_{s}\left(x_{s}\right)+\frac{\delta }{2}\|\;A_{1}x_{1}^{\left(k+1\right)}+\cdots +A_{s-1}x_{s-1}^{\left(k+1\right)}+A_{s}x_{s}+By^{\left(k\right)}+\frac{w^{\left(k\right)}}{\delta }{\|}_{2}^{2};\\ y^{\left(k+1\right)} & \in \text{arg}\;\min_{y}G\left(y\right)+\frac{\delta }{2}\|\;A_{1}x_{1}^{\left(k+1\right)}+\cdots +A_{s}x_{s}^{\left(k+1\right)}+By+\frac{w^{\left(k\right)}}{\delta }{\|}_{2}^{2};\\ w^{\left(k+1\right)} & =w^{\left(k\right)}+\delta (\sum_{i=1}^{s}A_{i}x_{i}^{\left(k+1\right)}+By^{\left(k+1\right)}). \end{array} \end{array}$$ (18)

The following lemma establishes the convergence result of ADMM with the nonconvex and nonsmooth objective [51].

**Lemma 1**. *Let* $F\left(x\right)=F_{1}\left(x_{1}\right)+\cdots +F_{s}\left(x_{s}\right)$ *and A* = [*A*_1_⋯*A*_*s*_]. *Suppose that the following assumptions S1–S5 hold, then the sequence generated by* Eq (18) *with any sufficiently large δ and any initialization has at least one limit point, and each limit point is a stationary point of* Eq (17).

*S1 (coercivity) Let* $D=\{\left(x,y\right)\in R^{N+L}:Ax+By=0\}$ *be the nonempty feasible set and* F(x)+G(y) *is coercive over* D;

*S2 (feasibility)* Im(*A*) ⊆ Im(*B*), *where* Im(⋅) *is defined as the image of a matrix*;

S3 (Lipschitz sub-minimization paths)

*(a) For any fixed x, there exists a Lipschitz continuous map* $G:\text{Im}\left(B\right)\to R^{L}$ *obeying* $G\left(u\right)=\text{arg}\;{\text{min}}_{y}\left\{F\left(x\right)+G\left(y\right):By=u\right\}$ *provided a unique minimizer*,

*(b) For i* = 1, ⋯, *s and fixed x*_−*i*_ *and y, there exists a Lipschitz continuous map* $F_{i}:\text{Im}\left(A_{i}\right)\to R^{n_{i}}$ *obeying* $F_{i}\left(u\right)=\text{arg}\;{\text{min}}_{x_{i}}\left\{F\left(x\right)+G\left(y\right):A_{i}x_{i}=u\right\}$ *provided a unique minimizer, where x*_−*i*_ ≔ [*x*_1_;⋯;*x*_*i*−1_;*x*_*i*+1_;⋯;*x*_*s*_];

*S4 (objective*-F *regularity)* F *is lower semi-continuous or* $F_{1}$ *is lower semi-continuous and* $F_{i},i=2,\cdots ,s$ *is restricted prox-regular*;

*S5 (objective*-G *regularity*) G *is Lipschitz differentiable with the constant* $L_{\nabla_{G}}>0$.

### 2.4 Iteratively reweighted least squares algorithm

IRLS algorithm [52, 53] solves the following nonconvex *ℓ*_*p*_ norm sparsity problem:

$$\begin{array}{c} \underset{z}{\text{min}}\{\frac{1}{2}\|z-\overset{˚}{z}{\|}_{2}^{2}+\lambda \|z{\|}_{p}^{p}\}, \end{array}$$ (19)

where $z,\overset{˚}{z}\in R^{n^{2}}$, and 0 < *p* < 1 causes the model to be nonconvex. It is showed that the IRLS is guaranteed to converge to its local minima with better theoretical and practical abilities than the iteratively reweighted *ℓ*_1_ (IRL1) [52–55]. Then, the problem Eq (19) can be approximated to

$$\begin{array}{c} \underset{z}{\text{min}}\{\frac{1}{2}\|z-\overset{˚}{z}{\|}_{2}^{2}+\sum_{i=1}^{n^{2}}\omega_{i}z_{i}^{2}\} \end{array}$$ (20)

with the weight $\omega_{i}=\lambda p(z_{i}^{2}+\epsilon {)}^{p/2-1}$ and *ϵ* ≪ 1 being a small positive number to avoid division by zero. Given *k*-th estimation $z^{\left(k\right)}=(z_{1}^{\left(k\right)},\cdots ,z_{n^{2}}^{\left(k\right)})$, IRLS first calculates the weight $\omega^{\left(k\right)}=(\omega_{1}^{\left(k\right)},\cdots ,\omega_{n^{2}}^{\left(k\right)})$, then updates *z* by solving the following problem:

$$\begin{array}{c} z^{\left(k+1\right)}=\text{arg}\;\underset{z}{\text{min}}\{\frac{1}{2}\|z-\overset{˚}{z}{\|}_{2}^{2}+\sum_{i=1}^{n^{2}}\omega_{i}^{\left(k\right)}z_{i}^{2}\}. \end{array}$$ (21)

We summarize the IRLS method in Algorithm 2.

**Algorithm 2** IRLS algorithm for minimizing Eq (19)

**input** $\overset{˚}{z}\in R^{n^{2}},p\in \left(0,1\right),\lambda >0,\epsilon ⪡1$.

**initialize** $z^{\left(0\right)}=\overset{˚}{z},k=0$.

**while** not converged **do**

 1: update weight: *ω*^(*k*)^ = λ*p*((*z*^(*k*)^)^2^ + *ϵ*)^*p*/2−1^;

 2: update *z*: $z^{\left(k+1\right)}=(I_{n^{2}}+\text{diag}\left(\omega^{\left(k\right)}\right){)}^{-1}\overset{˚}{z}$;

 3: *k* = *k* + 1;

**end while**

## 3 Proposed model and optimization method

In this section, we first present the proposed model and then solve it in the framework of nonconvex and nonsmooth ADMM.

### 3.1 Model formulation

Considering the advantages of the overlapping group sparse total variation and the high-order nonconvex total variation, we investigate the Poisson noisy image restoration problem with the following regularizer:

$$\begin{array}{c} \phi \left(f\right)=\phi_{\text{TO}}\left(f\right)+\eta \phi_{\text{HTVp}}\left(f\right), \end{array}$$ (22)

where *ϕ*_TO_(*f*) is the TVOGS term, $\phi_{\text{HTVp}}\left(f\right)≔\|(\nabla^{2}f){\|}_{p}^{p}=\sum_{i,j=1}^{n}\|{\left(\nabla^{2}f\right)}_{i,j}{\|}_{p}^{p}$ (0 < *p* < 1) is the nonconvex second-order TV term, and *η* > 0 is a regularization parameter. Substituting the hybrid regularizer Eq (22) into Eq (3) with the consideration to Eq (11), we obtain

$$\begin{array}{c} \underset{f}{\text{min}}\{\lambda D_{\text{KL}}\left(Hf||g\right)+\phi_{O}\left(\nabla f\right)+\eta \|\nabla^{2}f{\|}_{p}^{p}\}. \end{array}$$ (23)

In the model above, λ and *η* are used to control the data fidelity term and the nonconvex second-order regularizer, respectively. The data fidelity term is an ill-conditioned non-quadratic log-likelihood, while the regularizer is nonconvex and nonsmooth due to the presence of the *ℓ*_*p*_(0 < *p* < 1) quasi norm. This may increase the difficulty in minimizing the model. To circumvent this difficulty, we propose an efficient algorithm based on the framework of nonconvex and nonsmooth ADMM in the following subsection.

### 3.2 Optimization

In order to apply the variable splitting scheme, we introduce three auxiliary variables to transform the original complicated minimization problem into the following equivalent linear constraint minimization problem:

$$\begin{array}{c} \begin{array}{cc} \underset{f}{\text{min}}\lambda D_{\text{KL}}( & x_{1}\left||g\right)+\phi_{O}\left(x_{2}\right)+\eta \|x_{3}{\|}_{p}^{p}\\ \text{subject}\;\text{to}\; & x_{1}=Hf,x_{2}=\nabla f,x_{3}=\nabla^{2}f. \end{array} \end{array}$$ (24)

We establish a corresponding relation between the variables to conform with the ADMM framework Eq (16), as shown below:

(1) $x_{1}=Hf\in R^{n^{2}},x_{2}=\nabla f\in R^{2n^{2}},x_{3}=\nabla^{2}f\in R^{4n^{2}},y=f\in R^{n^{2}};$
(2) $F_{1}\left(x_{1}\right)=\lambda D_{\text{KL}}(x_{1}||g),F_{2}\left(x_{2}\right)=\phi_{O}\left(x_{2}\right),F_{3}\left(x_{3}\right)=\eta \|x_{3}{\|}_{p}^{p},G\left(y\right)=0;$
(3) $A_{1}=(\begin{array}{c} -I_{n^{2}}\\ 0_{3}^{T}\\ 0_{2} \end{array})$, $A_{2}=(\begin{array}{c} 0_{3}\\ -I_{2n^{2}}\\ 0_{1}^{T} \end{array})$, $A_{3}=(\begin{array}{c} 0_{2}^{T}\\ 0_{1}\\ -I_{4n^{2}} \end{array})$, $B=(\begin{array}{c} H\\ \nabla \\ \nabla^{2} \end{array})$,

where **0**_1_, **0**_2_, **0**_3_ denote zero matrix of size 2*n*^2^ × 4*n*^2^, 4*n*^2^ × *n*^2^, *n*^2^ × 2*n*^2^, respectively, and $I_{n^{2}}$ is the identity matrix of order *n*^2^. By introducing the Lagrangian multiplier $w=\left[w_{1};w_{2};w_{3}\right]\in R^{7n^{2}}$ (here $w_{1}\in R^{n^{2}},w_{2}\in R^{2n^{2}},w_{3}\in R^{4n^{2}}$) and the positive penalty parameter vector $\delta ={\left(\delta_{1},\delta_{2},\delta_{3}\right)}^{T}\in R^{3}$, we can turn Eq (24) into the unconstrained minimization of the following augmented Lagrangian function:

$$\begin{array}{c} \begin{array}{cc} L_{\delta }(x_{1}, & x_{2},x_{3},f;w)=\lambda D_{\text{KL}}(x_{1}\left||g\right)+\phi_{O}\left(x_{2}\right)+\eta \|x_{3}{\|}_{p}^{p}+\\ + & \frac{\delta_{1}}{2}\|x_{1}-Hf+\frac{w_{1}}{\delta_{1}}{\|}_{2}^{2}+\frac{\delta_{2}}{2}\|x_{2}-\nabla f+\frac{w_{2}}{\delta_{2}}{\|}_{2}^{2}+\frac{\delta_{3}}{2}\|x_{3}-\nabla^{2}f+\frac{w_{3}}{\delta_{3}}{\|}_{2}^{2}. \end{array} \end{array}$$ (25)

Then, the ADMM for solving Eq (24) starts its iteration to update every variable by minimizing Eq (25) alternatively. This iterative scheme could be split into several subproblems.

#### 3.2.1 *x*_1_-subproblem

According to the ADMM scheme, pulling out the terms with *x*_1_ from Eq (25) yields

$$\begin{array}{c} x_{1}^{\left(k+1\right)}=\text{arg}\;\min_{x_{1}}\{\lambda D_{\text{KL}}(x_{1}\left||g\right)+\frac{\delta_{1}}{2}\|x_{1}-Hf^{\left(k\right)}+\frac{w_{1}^{\left(k\right)}}{\delta_{1}}{\|}_{2}^{2}\}. \end{array}$$ (26)

It is apparent that each component of *x*_1_ can be solved separately. Through the basic mathematical manipulations in the case of *b* = 0, we obtain

$$\begin{array}{c} (x_{1}^{\left(k+1\right)}{)}_{i,j}=\frac{1}{2}[(Hf^{\left(k\right)}-\frac{w_{1}^{\left(k\right)}+\lambda }{\delta_{1}}{)}_{i,j}+\sqrt{(Hf^{\left(k\right)}-\frac{w_{1}^{\left(k\right)}+\lambda }{\delta_{1}}{)}_{i,j}^{2}+\frac{4\lambda g_{i,j}}{\delta_{1}}}]. \end{array}$$ (27)

#### 3.2.2 *x*_2_-subproblem

Minimizing $L_{\delta }\left(x_{1},x_{2},x_{3},f;w\right)$ with respect to *x*_2_ leads to the overlapping group sparse problem

$$\begin{array}{c} x_{2}^{\left(k+1\right)}=\text{arg}\;\min_{x_{2}}\{\frac{\delta_{2}}{2}\|x_{2}-(\nabla f^{\left(k\right)}-\frac{w_{2}^{\left(k\right)}}{\delta_{2}}){\|}_{2}^{2}+\phi_{O}(x_{2})\}. \end{array}$$ (28)

It is easy to see that this minimization problem matches the framework of Eq (12), thus *x*_2_ can be solved by Algorithm 1.

#### 3.2.3 *x*_3_-subproblem

By omitting terms irrespective of *x*_3_, we have

$$\begin{array}{c} x_{3}^{\left(k+1\right)}=\text{arg}\;\min_{x_{3}}\{\frac{\delta_{3}}{2}\|x_{3}-(\nabla^{2}f^{\left(k\right)}-\frac{w_{3}^{\left(k\right)}}{\delta_{3}}){\|}_{2}^{2}+\eta \|x_{3}{\|}_{p}^{p}\}. \end{array}$$ (29)

Since this problem involves the nonconvex *ℓ*_*p*_ norm, minimizing *x*_3_ is not straightforward. But, after some simple modifications, we can apply the IRLS algorithm according to Algorithm 2.

#### 3.2.4 *f*-subproblem

Considering Eq (25) with respect to *f*, we have

$$\begin{array}{c} \begin{array}{cc} f^{\left(k+1\right)}=\text{arg}\;\min_{f}\{\frac{\delta_{1}}{2}\|x_{1}^{\left(k+1\right)}-Hf+\frac{w_{1}^{\left(k\right)}}{\delta_{1}}{\|}_{2}^{2}+ & \frac{\delta_{2}}{2}\|x_{2}^{\left(k+1\right)}-\nabla f+\frac{w_{2}^{\left(k\right)}}{\delta_{2}}{\|}_{2}^{2}+\\ + & \frac{\delta_{3}}{2}\|x_{3}^{\left(k+1\right)}-\nabla^{2}f+\frac{w_{3}^{\left(k\right)}}{\delta_{3}}{\|}_{2}^{2}\}, \end{array} \end{array}$$ (30)

which can be solved by the following normal equation

$$\begin{array}{c} \begin{array}{cc} \left(\delta_{1}H^{T}H+\delta_{2}\nabla^{T}\nabla +\delta_{3}{\nabla^{2}}^{T}\nabla^{2}\right) & f=\delta_{1}H^{T}(x_{1}^{\left(k+1\right)}+\frac{w_{1}^{\left(k\right)}}{\delta_{1}})+\\ +\delta_{2}\nabla^{T} & (x_{2}^{\left(k+1\right)}+\frac{w_{2}^{\left(k\right)}}{\delta_{2}})+\delta_{3}{\nabla^{2}}^{T}(x_{3}^{\left(k+1\right)}+\frac{w_{3}^{\left(k\right)}}{\delta_{3}}). \end{array} \end{array}$$ (31)

We assume that both the image and convolution matrix are periodically extended, thus well-known fast Fourier transform can be adopted to efficiently solve Eq (31) [4, 56, 57], which results in an optimal solution

$$\begin{array}{c} f^{\left(k+1\right)}=F^{-1}(\frac{D}{C}) \end{array}$$ (32)

with

$$\begin{array}{c} \begin{array}{c} \;C=\delta_{1}F^{*}\left(H\right)\circ F\left(H\right)+\delta_{2}F^{*}\left(\nabla \right)\circ F\left(\nabla \right)+\delta_{3}F^{*}\left(\nabla^{2}\right)\circ F\left(\nabla^{2}\right),\\ D=F^{*}\left(H\right)\circ (\delta_{1}F\left(x_{1}^{\left(k+1\right)}\right)+F\left(w_{1}^{\left(k\right)}\right))+F^{*}\left(\nabla \right)\circ (\delta_{2}F\left(x_{2}^{\left(k+1\right)}\right)+F\left(w_{2}^{\left(k\right)}\right))+\\ \;+F^{*}\left(\nabla^{2}\right)\circ (\delta_{3}F\left(x_{3}^{\left(k+1\right)}\right)+F\left(w_{3}^{\left(k\right)}\right)), \end{array} \end{array}$$ (33)

where F denotes the FFT operator, * and ∘ stand for complex conjugate and element-wise multiplication, respectively. The division is computed by component-wise fashion. Note that many subterms need to be computed only once during the iterative updates.

#### 3.2.5 *w*-subproblem

Finally, the updating scheme of the Lagrangian multipliers can be written as

$$\begin{array}{c} \begin{array}{cc} w_{1}^{\left(k+1\right)} & =w_{1}^{\left(k\right)}+\delta_{1}(x_{1}^{\left(k+1\right)}-Hf^{\left(k+1\right)});\\ w_{2}^{\left(k+1\right)} & =w_{2}^{\left(k\right)}+\delta_{2}(x_{2}^{\left(k+1\right)}-\nabla f^{\left(k+1\right)});\\ w_{3}^{\left(k+1\right)} & =w_{3}^{\left(k\right)}+\delta_{3}(x_{3}^{\left(k+1\right)}-\nabla^{2}f^{\left(k+1\right)}). \end{array} \end{array}$$ (34)

In summary, the key steps of the ADMM algorithm for solving the suggested model are described in Algorithm 3.

**Algorithm 3** The ADMM algorithm for solving Eq (23)

**input** *p*, λ, *η*, *δ*, *K*, *N*_in_.

**initialize** *f*^(0)^, *k* = 0, *w*^(0)^ = **0**.

**while** a stopping criteria unsatisfied **do**             ▹ outer loop

 1: Update $x_{1}^{\left(k+1\right)}$ according to Eq (27).

 2: Update $x_{2}^{\left(k+1\right)}$ according to Eq (28) using Algorithm 1.

 3: Update $x_{3}^{\left(k+1\right)}$ according to Eq (29) using Algorithm 2.

 4: Update *f*^(*k*+1)^ according to Eq (32).

 5: Update $w_{i}^{\left(k+1\right)},i=1,2,3$ according to Eq (34).

 6: *k* = *k* + 1.

**end while**

We discuss the convergence of the proposed algorithm. Inspired by [51], by verifying the assumptions in Lemma 1, we can obtain the following convergence result for Algorithm 3.

**Theorem 1**. *The sequence of* (*x*_1_, *x*_2_, *x*_3_, *f*) *generated by Algorithm 3 with any sufficiently large δ and any start point will converge to a stationary point of the augmented Lagrangian* $L_{\delta }$.

*Proof*. Since our model fits the framework of Eq (16), it remains only to check S1–S5 in Lemma 1. It is easy to verify that *D*_KL_(*x*_1_||*g*) is coercive [58] as well as *ϕ*_O_(*x*_2_) and $\|\nabla^{2}x_{3}{\|}_{p}^{p}$. Thus, S1 holds. *Ax* + *By* = **0** implies S2. S3 holds because both *A* and *B* are full column rank matrices. *D*_KL_(*x*_1_,*g*) is lower semi-continuous and *ℓ*_*p*_-quasi norm is restricted prox-regular [51]. Furthermore, *ϕ*_O_(*x*_2_) is convex, hence restricted prox-regular. It is clear that S5 holds, which completes the proof.

The convergence of Algorithm 3 can also be verified experimentally.

## 4 Numerical experiments

In this section, we present the numerical experiments to illustrate the effectiveness of the proposed method for the Poisson noisy image restoration, compared with the closely related state-of-the-art methods including TVOGS [4], SAHTV [5], SB-FA [38] and FT-ADMM [37]. The TVOGS method introduced the overlapping group sparse TV prior combined with the box-constraint as a regularizer and solved the optimization model under the ADMM framework, but denoising is out of their consideration. In the SAHTV method, Liu et al. [5] combined the first and second-order TV priors, and updated their pixel-wise weighting coefficients with the local information during the consecutive estimation of the latent image. FT-ADMM, whose regularizer is the ℓ_0_ norm of the wavelet representation of the image, was also proposed to efficiently eliminate the staircase artifacts. Fang and Yan [38] proposed to combine the ℓ_1_ norm of the framelet representation of the latent image with the TV prior, and solved the resultant model by the split Bregman method. Since they recommended using the SB-FA algorithm without the TV prior through the numerical experiments, we adopt SB-FA as a competitive method rather than SB-FATV with the TV prior.

All experiments in this paper are implemented on Windows 10 64-bit and Matlab R2016b running on a desktop computer with an Intel Core i5-4590 CPU 3.3GHz and 8GB of RAM. We uploaded the code for our algorithm on https://github.com/KSJhon/PoissonDeblur_hybrid.

**Fig 1:**
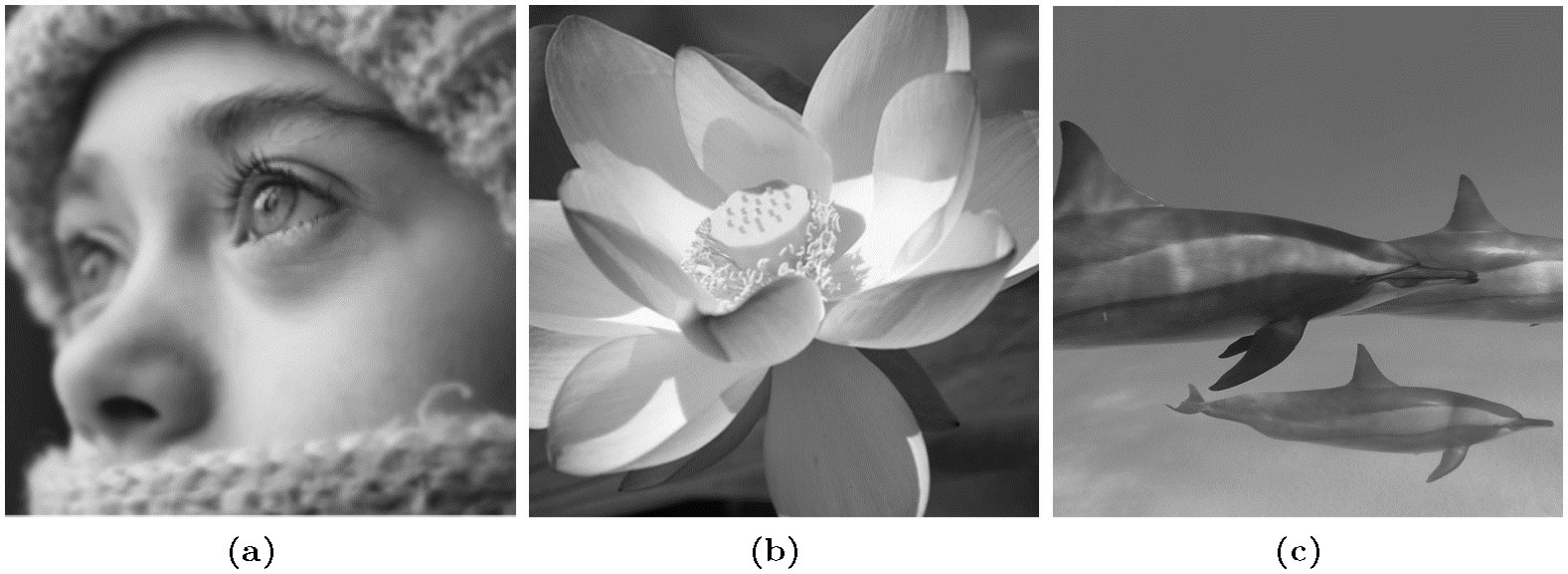
Test images used in our experiments. (a): “beauty” (256 × 256). This image was republished from https://www.flickr.com/photos/90471071@N00/3201337190 under a CC BY license, with permission from Irina Gheorghita, original copyright 2009; (b): “lotus” (500 × 500). This image was republished from https://www.flickr.com/photos/robertlylebolton/7797693474 under a CC BY license, with permission from Robert Lyle Bolton, original copyright 2012; (c): “dolphin” (512 × 512). This image was republished from https://www.flickr.com/photos/grovecrest/6981622176 under a CC BY license, with permission from Simon Lewis, original copyright 2011.

To proceed with the simulation experiments, we intentionally degrade the clean image to construct the corrupted image. More specifically, an original clean image is first scaled to a preset peak value MAX_*f*_, which decides the different levels of Poisson noise. After that, the scaled image is convolved with a given PSF to simulate the blurring effect, and further contaminated by the signal-dependent Poisson noise. In the following experiments, we set MAX_*f*_ to be 100, 200, 300, and 350, in which the lower MAX_*f*_ indicates the relatively higher noise level. For the deblurring simulations, we consider three types of blur kernels: (1) a 9 × 9 Gaussian kernel with standard deviation 1; (2) a linear motion blur with a motion length 5 and an angle 45° in the counterclockwise direction; (3) a kernel from Levin et al.’s public dataset [59]. All blurring operations are fulfilled by the Matlab built-in function “fspecial”, and the Poisson noise is generated by “poissrnd” without any additional parameter. The test images with various sizes are shown in Fig 1.

We quantitatively evaluate the performances of the proposed method and the competing methods by means of the peak signal-to-noise ratio (PSNR) and the structural similarity (SSIM), defined as

$$\begin{array}{c} \text{PSNR}=20\;\text{log}\frac{n{\text{MAX}}_{f}}{\|f-\hat{f}{\|}_{2}}, \end{array}$$ (35)

$$\begin{array}{c} \text{SSIM}=\frac{\left(2\mu_{f}\mu_{\hat{f}}+C_{1}\right)\left(2\sigma_{f,\hat{f}}+C_{2}\right)}{\left(\mu_{f}^{2}+\mu_{\hat{f}}^{2}+C_{1}\right)\left(\sigma_{f}^{2}+\sigma_{\hat{f}}^{2}+C_{2}\right)}, \end{array}$$ (36)

where *f* is the original image, $\hat{f}$ is the restored image, $\mu_{f},\mu_{\hat{f}}$ are the respective averages, $\sigma_{f}^{2},\sigma_{\hat{f}}^{2}$ are the respective variances, $\sigma_{f,\hat{f}}$ is the covariance of *f* and $\hat{f}$, and $C_{1}={\text{MAX}}_{f}^{2}/10^{4},C_{2}=9\cdot {\text{MAX}}_{f}^{2}/10^{4}$ by default. Generally, the larger the PSNR and the closer SSIM to 1, the better the quality of the restored image. In the experiments, we terminate the iteration in Algorithm 3 when the relative error (RelErr) between two consecutive estimates falls below the predefined tolerance level *ε*, as follows:

$$\begin{array}{c} \frac{\|f^{\left(k+1\right)}-f^{\left(k\right)}{\|}_{2}}{\|f^{\left(k+1\right)}{\|}_{2}}<\varepsilon . \end{array}$$ (37)

### 4.1 Selection of parameters

Since the convergence of the nonconvex ADMM and its suboptimization in Algorithm 3 depends on the values of parameters, they require careful tuning.

For the choice of *p*, as in the experiments reported in [40], we set it to be 0.1 throughout the experiments, with the consideration of the important aspect of *ℓ*_*p*_ norm, which says that taking *p* sufficiently close to 0 favors to preserve sharp edges in the restored image.

**Fig 2:**
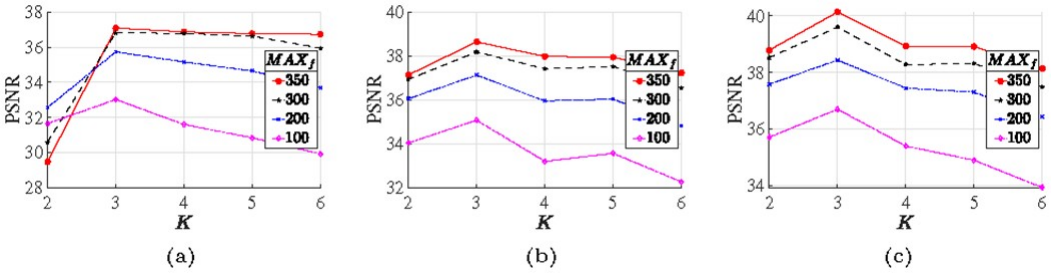
Evolution of PSNR values of denoised images with MAX_*f*_ = 350, 300, 200 and 100 along the varying group sizes. (a): “beauty” image; (b): “lotus” image; (c): “dolphin” image.

The group size *K* also plays an important role in balancing the trade-off between the global noise filtering performance and the computational cost. In order to find the optimal *K*, we conduct a denoising experiment by varying *K* while keeping other parameters remained, as shown in Fig 2. Obviously, *K* = 3 is the best choice for all noise levels, so we fix *K* = 3 in the following experiments. In addition, the number of inner iterations, *N*_*in*_, also affects the MM algorithm. We straightforwardly fix *N*_*in*_ = 5 as indicated in [4, 29, 39, 40]. To balance the accuracy and speed, we empirically set *N*_*out*_ = 50, *ε* = 10^−3^. The other parameters, including the regularization parameters (λ, *η*) and penalty parameter *δ*, are manually adjusted to achieve the highest improvement in the PSNR and SSIM values. In the following subsections, we compare the proposed method (named HTVp-OGS) with the state-of-the-art methods for denoising and deblurring images under the Poisson noise. Unless otherwise indicated, all parameters involved in the competitive algorithms are carefully selected near the given default values to give the best performance, respectively.

### 4.2 Denoising

Here, we show that our model provides better results in Poisson noise removal through the comparison with the state-of-the-art methods: SB-FA [38], SAHTV [5], and FT-ADMM [37].

**Fig 3:**
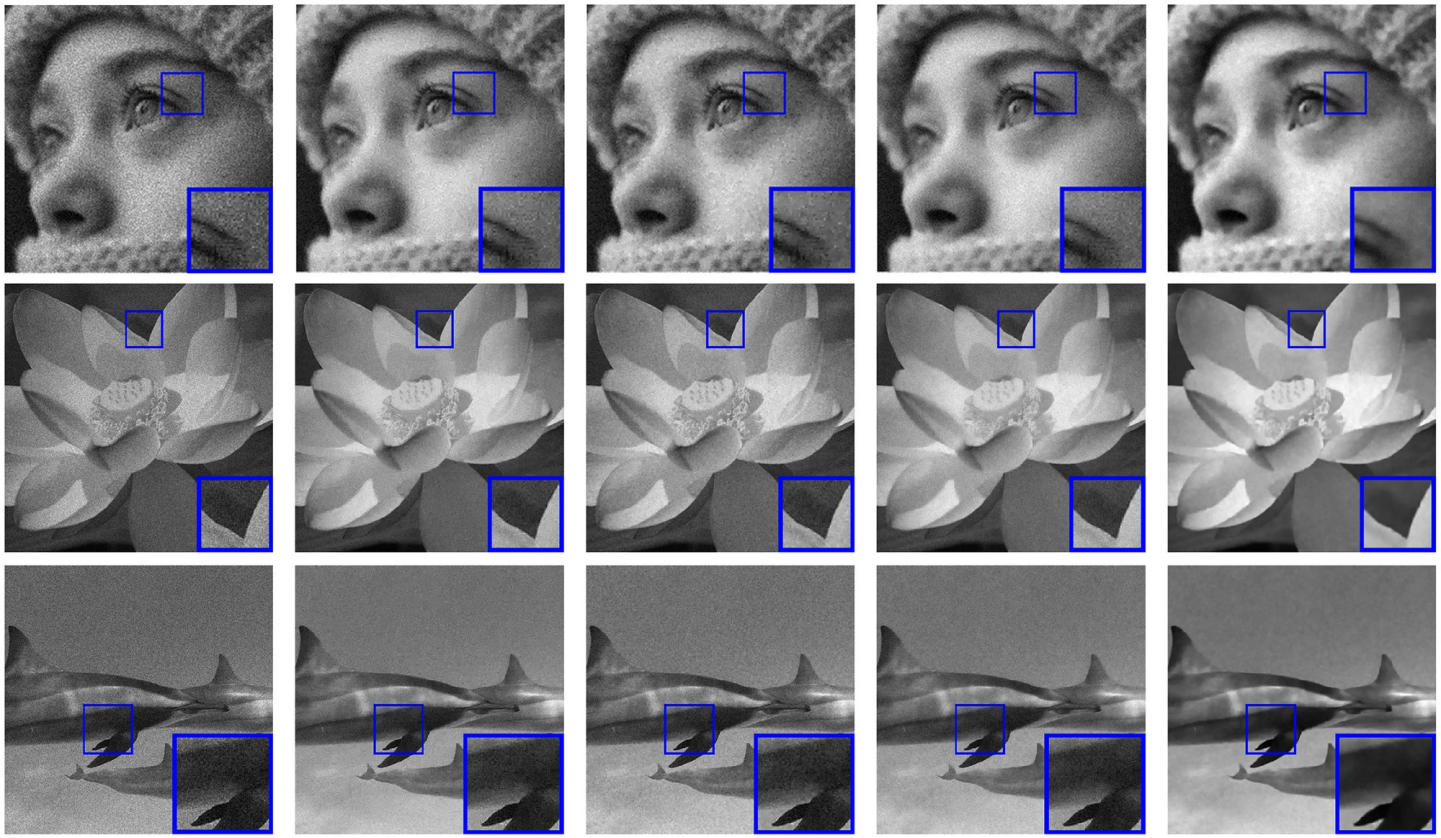
Denoised images and a zoom-in region from the Poisson noisy images with the noise level MAX_*f*_ = 100. first column: Poisson noisy images; second column: denoised images by SB-FA [38]; third column: denoised images by SAHTV [5]; fourth column: denoised images by FT-ADMM [37]; fifth column: denoised images by HTVp-OGS.

To evaluate the denoising performance, we just set *H* as an identity matrix, and empirically fix *δ* = (5 ⋅ 10^−3^, 3 ⋅ 10^−2^, 2 ⋅ 10^−4^)^*T*^. We can observe that λ = 3 ⋅ MAX_*f*_ can bring the satisfactory results of Eq (23). The other regularization parameter *η* is hand-tuned to get the best denoising performance according to the noise level. The denoising results, in terms of the PSNR and SSIM values, are shown in Table 1. From Table 1, it is obvious that the PSNR and SSIM values of the images denoised by our model are higher than those of the other three methods (SB-FA, SAHTV, FT-ADMM). We display in Fig 3. the denoised versions and the zoom-in regions obtained by our method and other competitive methods from the noisy images with the noise level MAX_*f*_ = 100.

**Table 1:** The PSNR and SSIM values for denoised images by different methods.

*MAX*_*f*_	clean(*f*)	noisy(*g*)	denoised($\hat{f}$)			
			SB-FA	SAHTV	FT-ADMM	HTVp-OGS
350	beauty	27.97/0.649	34.96/0.929	35.41/0.941	34.17/0.920	**37.08/0.955**
	lotus	28.12/0.530	36.34/0.913	37.42/0.936	34.96/0.898	**38.64/0.957**
	dolphin	27.95/0.465	38.18/0.926	38.41/0.944	36.75/0.910	**40.14/0.966**
300	beauty	27.31/0.620	34.29/0.912	34.77/0.931	33.73/0.906	**36.82/0.953**
	lotus	27.46/0.498	35.63/0.890	36.73/0.922	34.42/0.876	**38.18/0.955**
	dolphin	27.28/0.431	37.04/0.898	37.89/0.933	35.67/0.884	**39.61/0.964**
200	beauty	25.55/0.539	32.16/0.844	32.81/0.880	31.85/0.846	**35.73/0.944**
	lotus	25.70/0.412	33.23/0.796	34.08/0.843	32.38/0.786	**37.12/0.945**
	dolphin	25.52/0.348	33.94/0.787	35.00/0.855	33.06/0.779	**38.44/0.956**
100	beauty	22.53/0.402	27.74/0.645	28.06/0.676	28.18/0.660	**33.01/0.917**
	lotus	22.68/0.283	28.35/0.542	28.45/0.562	28.49/0.542	**35.06/0.925**
	dolphin	22.51/0.227	28.41/0.500	28.66/0.539	28.58/0.544	**36.70/0.939**

### 4.3 Deblurring

In our deblurring experiments, we consider three types of blur kernels. As mentioned above, the first two are artificial to respectively mimic the effect of the out-of-focus blur and motion blur, and the last one is the blur kernel from Levin et al.’s dataset [59]. For the sake of a fair comparison with other methods, we equally set the tolerance level *ε* = 10^−3^ and iteration number *N*_*out*_ = 50 in all experiments. As in the denoising experiments, we fix λ = 3 ⋅ MAX_*f*_ and select the penalty parameter *δ* as (0.01, 0.1, 0.01)^*T*^. The values of *δ* and λ are kept constant, and the other parameter *η* is selected by the grid search scheme to obtain high-quality restored images in the following three cases:

**Setting 1**. Gaussian blur case: We set *η* = 18, 14, 6, and 2 according to the noise level MAX_*f*_ = 350, 300, 200, and 100, respectively. The detailed comparison of five methods is provided in Table 2.
**Setting2**. linear motion blur case: We keep *δ* and λ remained and empirically set *η* = 8, 6, 4, 1, and the detailed results are summarized in Table 3.
**Setting 3**. ground truth blur case: In this case, we set *η* = 4, 3, 2, 0.2, and the detailed results are summaried in Table 4.

**Table 2:** The PSNR and SSIM values for the Poisson image restoration by different methods in the case of the Gaussian blur with kernel size 9 × 9 and variation 1.

MAX_*f*_	clean(*f*)	degraded(*g*)	restored($\hat{f}$)				
			SB-FA	SAHTV	TVOGS	FT-ADMM	HTVp-OGS
350	beauty	27.00/0.62	32.81/0.92	32.45/0.93	31.19/0.92	32.39/0.93	**32.85/0.94**
	lotus	27.68/0.51	35.82/0.92	36.31/0.94	34.64/0.93	34.90/0.93	**36.66/0.94**
	dolphin	27.55/0.45	36.70/0.93	36.97/0.94	36.67/0.95	35.87/0.95	**37.69/0.95**
300	beauty	26.47/0.59	32.58/0.92	32.19/0.92	31.16/0.92	32.33/0.93	**32.60/0.93**
	lotus	27.08/0.47	35.43/0.91	36.02/0.93	34.61/0.93	34.84/0.93	**36.37/0.94**
	dolphin	26.93/0.41	36.09/0.92	36.64/0.94	36.50/0.95	35.83/0.94	**37.37/0.95**
200	beauty	24.95/0.51	31.51/0.88	31.41/0.90	31.02/0.91	**31.96**/0.91	31.92/**0.92**
	lotus	25.44/0.39	33.98/0.87	34.96/0.91	34.36/0.92	34.50/0.92	**35.48/0.93**
	dolphin	25.27/0.33	34.49/0.87	35.67/0.92	35.99/0.94	35.66/0.93	**36.58/0.95**
100	beauty	22.22/0.37	28.50/0.75	29.78/0.85	30.42/0.90	30.66/0.87	**30.57/0.90**
	lotus	22.55/0.27	29.98/0.70	32.44/0.84	33.28/0.90	32.86/0.85	**33.87/0.92**
	dolphin	22.39/0.22	30.28/0.68	33.19/0.85	34.53/0.91	33.78/0.86	**35.29/0.94**

**Table 3:** The PSNR and SSIM values for the Poisson image restoration by different methods in the case of the motion blur with length 5 and angle 45°.

MAX_*f*_	clean(*f*)	degraded(*g*)	restored($\hat{f}$)				
			SB-FA	SAHTV	TVOGS	FT-ADMM	HTVp-OGS
350	beauty	26.64/0.61	31.34/0.92	31.90/0.92	29.96/0.90	31.76/0.92	**32.33/0.93**
	lotus	27.54/0.50	34.22/0.93	35.62/0.93	33.39/0.92	34.56/0.93	**36.31/0.94**
	dolphin	27.45/0.44	36.07/0.94	37.06/0.94	35.63/0.94	35.62/0.95	**37.68/0.95**
300	beauty	26.13/0.58	31.32/0.92	31.71/0.92	29.98/0.90	31.58/0.92	**32.12/0.93**
	lotus	26.94/0.47	34.21/0.92	35.33/0.93	33.39/0.92	34.27/0.93	**36.04/0.94**
	dolphin	26.85/0.41	36.01/0.94	36.72/0.94	35.54/0.94	35.49/0.93	**37.46/0.95**
200	beauty	24.74/0.50	31.11/0.91	30.97/0.90	29.95/0.90	31.12/0.90	**31.56/0.92**
	lotus	25.34/0.39	34.08/0.92	34.57/0.92	33.24/0.91	33.79/0.91	**35.32/0.93**
	dolphin	25.23/0.33	35.78/0.94	35.88/0.93	35.19/0.94	35.19/0.92	**36.58/0.95**
100	beauty	22.10/0.37	**30.39**/0.88	29.88/0.87	29.67/0.89	29.82/0.84	30.37/**0.90**
	lotus	22.79/0.34	31.48/0.84	31.51/0.83	31.17/0.84	30.77/0.80	**31.79/0.85**
	dolphin	22.37/0.21	34.56/0.91	33.81/0.88	33.92/0.91	33.23/0.84	**35.34/0.94**

**Table 4:** The PSNR and SSIM values for the Poisson image restoration by different methods in the case of the ground truth blur.

MAX_*f*_	clean(*f*)	degraded(*g*)	restored($\hat{f}$)				
			SB-FA	SAHTV	TVOGS	FT-ADMM	HTVp-OGS
350	beauty	25.44/0.56	29.81/0.89	30.96/0.90	29.22/0.87	30.78/0.91	**31.17/0.91**
	lotus	26.49/0.46	32.69/0.91	34.78/0.92	32.70/0.91	33.27/0.92	**34.95/0.93**
	dolphin	26.83/0.42	34.58/0.94	35.84/0.94	34.85/0.94	34.38/0.94	**36.43/0.95**
300	beauty	25.06/0.53	29.80/0.89	30.74/0.90	29.22/0.87	30.66/0.90	**30.97/0.91**
	lotus	26.03/0.43	32.66/0.91	34.45/0.92	32.67/0.91	33.24/0.91	**34.62/0.92**
	dolphin	26.31/0.39	34.47/0.94	35.57/0.93	34.79/0.94	34.38/0.94	**36.18/0.94**
200	beauty	23.93/0.46	29.72/0.88	30.01/0.88	29.21/0.87	30.27/0.88	**30.31/0.89**
	lotus	24.69/0.36	32.67/0.91	33.49/0.90	32.51/0.90	33.01/0.90	**33.90/0.92**
	dolphin	24.84/0.31	34.42/0.93	34.67/0.91	34.54/0.93	34.43/0.93	**35.22/0.94**
100	beauty	21.62/0.33	**29.30**/0.87	28.74/0.83	28.96/0.87	29.26/0.84	29.20/**0.87**
	lotus	22.14/0.24	32.19/0.89	31.43/0.84	31.91/0.89	32.04/0.86	**32.55/0.90**
	dolphin	22.17/0.20	33.77/0.92	32.19/0.86	33.57/0.91	33.42/0.88	**34.18/0.93**

**Fig 4:**
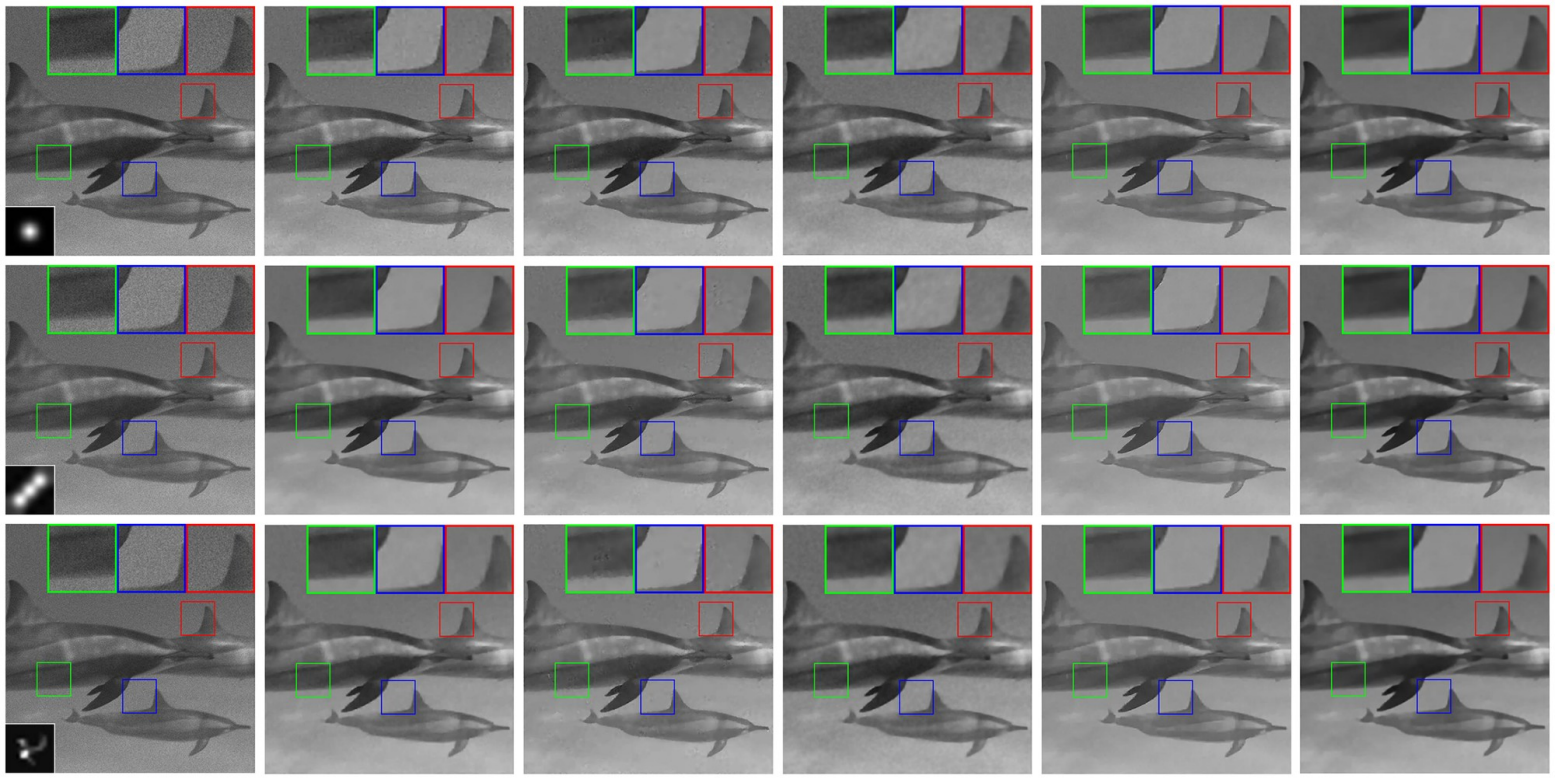
Restored “dolphin” images and zoom-in regions from the degraded images with different blur kernels and the Poisson noise of level MAX_*f*_ = 200. first row: Gaussian blur; second row: motion blur; third row: ground truth blur; first column: degraded images (PSF is shown at the bottom-left corner); second column: restored images by SB-FA [38]; third column: restored images by SAHTV [5]; fourth column: restored images by TVOGS [4]; fifth column: restored images by FT-ADMM [37]; sixth column: restored images by HTVp-OGS.

**Fig 5:**
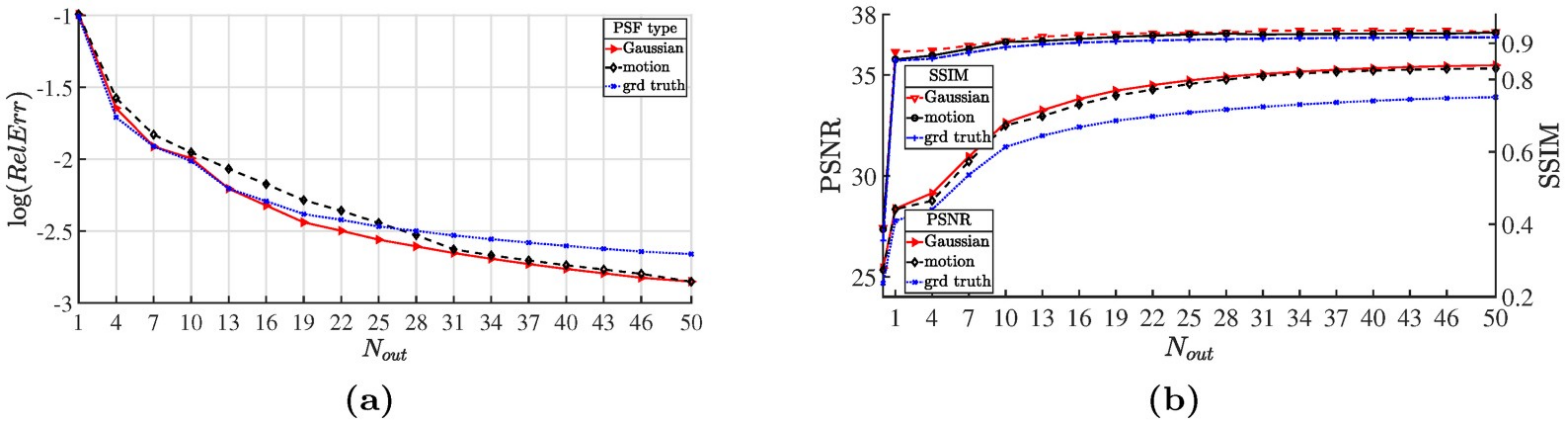
Plot of performance along the HTVp-OGS iterations for restoring “lotus” images blurred by different kernels and corrupted by the Poisson noise of level MAX_*f*_ = 200. (a) relative error in log scale; (b) PSNR and SSIM values.

In Fig 4, we show the degraded “dolphin” images which are blurred by the corresponding kernels and further corrupted by the Poisson noise of noise level 200, as well as its restored versions obtained through our method and the competitive methods. Fig 5(a) depicts the RelErr curves for the sequence of temporary estimates of the “lotus” image obtained during the HTVp-OGS iterations. Besides, in Fig 5(b), we show that, as the iteration proceeds, the PSNR and SSIM values moderately increase. From the results of the above three experiments, we can see that in most cases, our method provides the best results in terms of the PSNR and SSIM values, and in extreme cases, we obtain a slightly lower PSNR value than others, but the SSIM value still keeps the best. It is worth noting that the transform-based methods (SB-FA and FT-ADMM) are quite time-consuming because they inherently involve some complicated nonlinear operations [3], while our method works in the gradient domain, making it take a relatively short running time.

## 5 Conclusions

In this paper, we propose a new method to restore Poissonian images (including denoising and deblurring) by using a hybrid regularizer, which combines the overlapping group sparse total variation with the high-order nonconvex total variation. This regularizer allows us to exploit their advantages in order to alleviate the staircase artifacts while preserving the original sharp edges simultaneously. The model derived from the Bayesian perspective is effectively solved by the nonconvex and nonsmooth ADMM optimization framework. We adopt the MM and IRLS algorithm for solving the subproblems accompanied by the OGS and nonconvex second-order total variation priors, respectively. Numerical experiments demonstrate that the proposed method outperforms the other related methods in terms of the PSNR and SSIM values. The study on adaptively determining the optimal parameters according to the image content and noise level will be the future work.

## Supporting information

S1 File (STY)
